# Interplay between innate immune cells and inflammatory mediators in dengue infection: an evolving therapeutic paradigm

**DOI:** 10.1017/erm.2025.10021

**Published:** 2025-09-23

**Authors:** Akrite Mishra, Sudeshna Mallik, Pritam Saha, Sankhanil Dhara, Sumi Mukhopadhyay

**Affiliations:** 1 Department of Laboratory Medicine, Calcutta School of Tropical Medicine, Kolkata, India; 2 Department of Tropical Medicine, Calcutta School of Tropical Medicine, Kolkata, India

**Keywords:** chemokines, cytokines, dengue virus, innate immunity, therapeutics

## Abstract

**Background:**

Dengue is one of the neglected tropical diseases endemic to tropical and subtropical regions worldwide. Due to its substantial disease burden, this arthropod-borne viral disease is a significant public health concern. Infection involving any one of the five distinct serotypes causes a wide range of disease manifestations, from self-limiting to mild to life-threatening outcomes.

**Methods:**

The current review comprehensively provides an overview of dengue virus-mediated immunopathogenesis with special emphasis on innate immune cells, their pathogen recognition sensors and their association with pathogenesis. Additionally we have also briefly discussed recent advancements in vaccine studies and the development of therapeutics over the last decade.

**Results:**

The immunological response to dengue virus involves an amalgamation of a variety of innate cells and inflammatory mediators, resulting in the favouring or dampening of the antiviral response. Viral components activating innate cells through pattern recognition receptors, such as Toll-like receptors, retinoic-acid-inducible gene I and melanoma differentiation-associated gene 5, are vital in eliciting a downstream signalling cascade, which culminates in the secretion of inflammatory proteins.

**Conclusion:**

Understanding the specific mechanisms involved in the acute phase of infection is indispensable for detecting differential biomarkers against flavivirus infections as well as designing more efficient therapeutic agents and vaccines.

## Introduction

Dengue virus (DENV) is a positive-sense single-stranded ribonucleic acid (RNA) virus belonging to the member of the Flaviviridae family that triggers vector-borne infections. It is transmitted to humans by female mosquitoes of the species *Aedes aegypti*, and in some infrequent cases by *Aedes albopictus*, which are most often found in tropical and subtropical regions (Refs 1, 2). The viral genome entering the host cell synthesizes structural (capsid, membrane and envelope) and non-structural proteins (NS1, NS2A, NS2B, NS3, NS4A, NS4B and NS5). These non-structural proteins assist in viral replication, while the structural protein, especially the envelope protein, is involved in attachment and host cell receptor interaction for virus entry (Ref. 2). There are four antigenically varying serotypes, including DENV 1–DENV 4, which have been uncovered from various regions worldwide, and they exhibit strong immunological cross-reactivity due to a 65–70% sequence similarity (Ref. 3). Recently, a newly discovered strain, named DENV-5, was detected in a patient in Sarawak (Ref. 4). Infection with one serotype confers perpetual immunity against the same serotype.

An estimated 390 million individuals get infected annually, with 96 million exhibiting symptomatic cues of the disease, which is approximately three times the World Health Organization’s (WHO) original projection. India contributes approximately 34% to the world’s dengue burden (Ref. 5). In another 2019 study, the WHO designated 10 global health hazards, of which dengue was one of them (Ref. 6). These astounding figures make DENV one of the most prevalent infectious diseases globally circulated by arthropods. Dengue fever (DF) has become increasingly prevalent worldwide over the last two decades, posing a major threat to public wellness. From 2000 to 2019, the WHO reported a tenfold spike in cases reported globally, rising from 500,000 to 5.2 million in a span of two decades, with a sudden dip in cases on account of the underreporting during the active pandemic days. This was followed by a drastic reversal in 2023, with a surge in dengue cases, coinciding with the simultaneous occurrence of multiple outbreaks and cases diversifying into areas that were previously unaffected (Ref. 7). While DENV may be endemic in certain countries, healthcare professionals worldwide have reservations about the possibility that individuals commuting from non-endemic to dengue-affected areas might contract the virus, thereby aiding in the proliferation and establishment of local transmission in non-endemic zones (Ref. 8).

Dengue infection manifests as a mild febrile illness with symptoms such as fever, headache, muscle and/or bone pain and rash. A mild form of infection can transition to dengue with warning signs, which are characterized by abdominal pain, restlessness, hepatomegaly, vascular permeability and hypovolemia. Few cases can present as a more life-threatening form of the disease with potentially fatal outcomes, such as fluid accumulation, severe bleeding, impaired consciousness and organ impairment (Refs 3, 9, 10). In the acute phase, the innate immune system triggers a wide range of cells, resulting in the secretion of an array of inflammatory mediators in the microenvironment, affecting local and systemic viral replication. Uncontrolled activation of the innate immune cells that aid in the clearance of viral components leads to the exaggeration of immune responses, which culminates in severe conditions (Ref. 11). The current standard of care remains in palliative treatments; with increasing global burden, it necessitates improved control strategies comprising the development of effective vaccines and antiviral treatment methodologies (Ref. 3).

Several epidemiological studies have determined that a high DENV viremia corresponds to a higher possibility of developing severe circumstances (Ref. 12). Moreover, reinfection with a different serotype increases the likelihood of serious repercussions (Ref. 3). One of the factors associated with severe outcomes is antibody-dependent enhancement (ADE), which is primarily due to the presence of different serotypes. Additionally, conserved epitope sequences of other flaviviruses could also be a contributing factor (Ref. 13). The basic principle by which ADE occurs is when non-neutralizing and sub-neutralizing antibodies interact with immune cells and enhance viral entry and synthesis. Two types of ADE have been established, wherein intrinsic ADE aids in blocking Toll-like receptor (TLR) expression and signalling, eventually resulting in enhanced viral production, and extrinsic ADE enhances viral uptake (Ref. 14). A study conducted by Cui *et al.* reported that the pr4 epitope of the dengue viral genome influences the ADE pathway, hypothesizing that eliminating the epitope might be beneficial in reducing ADE, offering an innovative strategy in vaccine development (Ref. 15). Another study by Teo *et al.* proposed that afucosylated IgG1 enhanced the extrinsic ADE pathway, emphasizing their role in severe disease progression (Ref. 16). To further enhance vaccine research, more in-depth studies are necessary to understand the underlying mechanism of ADE.

Although significant advancements are in progress concerning anti-DENV research, regrettably, a clinically licensed antiviral therapy medication or vaccine remains to be discovered. For developing targeted dengue therapeutics, understanding the interplay between innate immune cells and soluble factors is essential. Compounds that modulate innate immune cells, signalling cascades or crucial immunomodulatory mediators can help mitigate the morbidity and mortality associated with severe dengue and might offer novel approaches towards therapeutics (Ref. 17).

**Figure 1.**
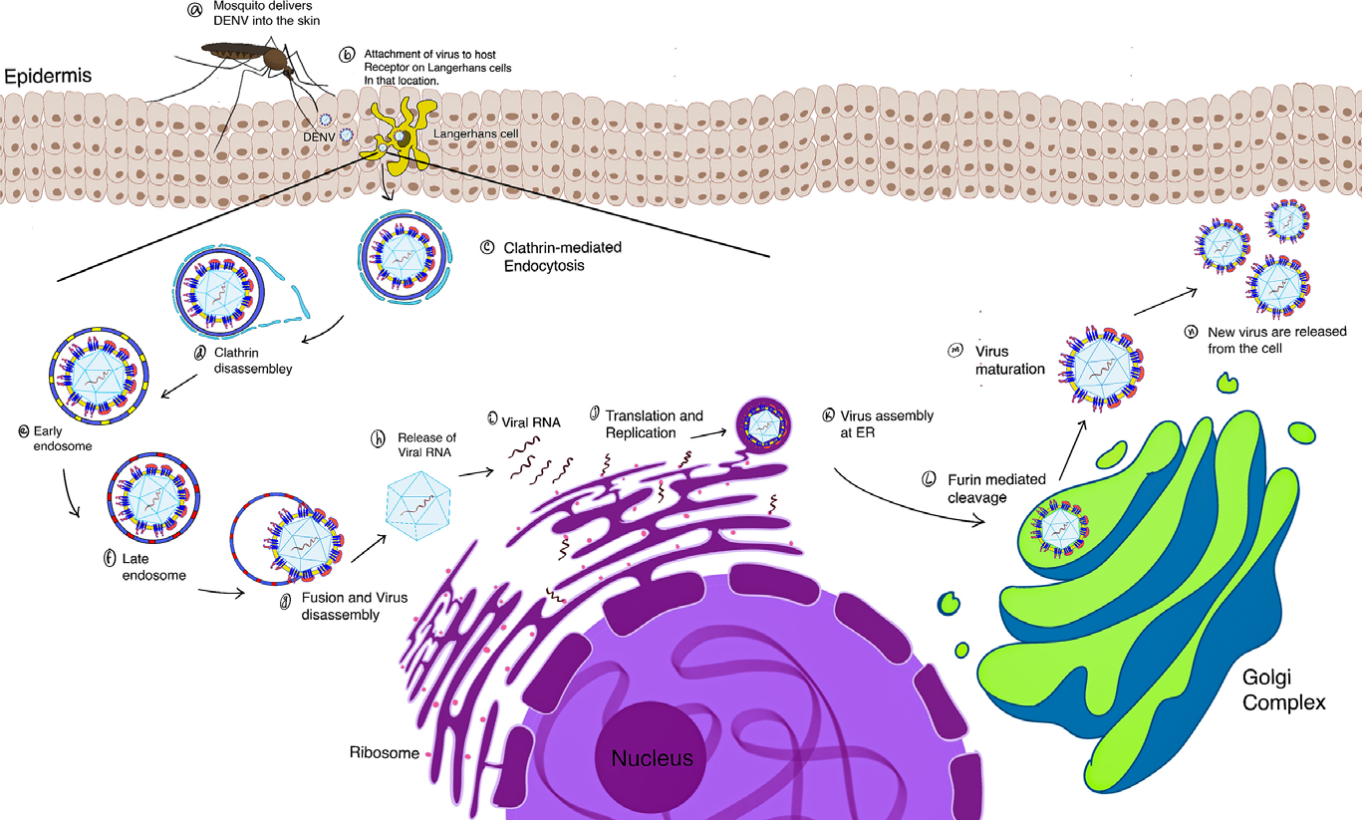
Schematic representation of the dengue virus (DENV) replication cycle. **(a–n)** After the mosquito bites, the viral particle in the epidermis and dermis induces interaction between the pathogen and migratory cells. DENV particles bind to host cell factors and then infiltrate the cell by clathrin-mediated endocytosis. With clathrin disassembly, endosomal processing begins, and it proceeds from the early endosome to the late endosome. Modulations inside the endosomal bubble result in fusion of the viral envelope and host membrane. This disassembly leads to the release of capsid-bound viral RNA in the cytoplasm. Viral replication and translation occur in the ER. Immature viral particle forms in the ER. Maturation of the viral particle proceeds in the trans-Golgi network by Furin-mediated cleavage. The mature viral particle is then released from the cell, completing its replication cycle, and is equipped to infect other cells.

In this review, we enhance our understanding of the disease pathophysiology by providing a general perspective of the interplay between the innate immune cells and inflammatory mediators post-DENV infection. Finally, it briefly discusses the contemporary approaches in target-based therapeutic research in dengue infection (Figure 1).

## Interplay between innate immune response and inflammatory mediators

**Figure 2.**
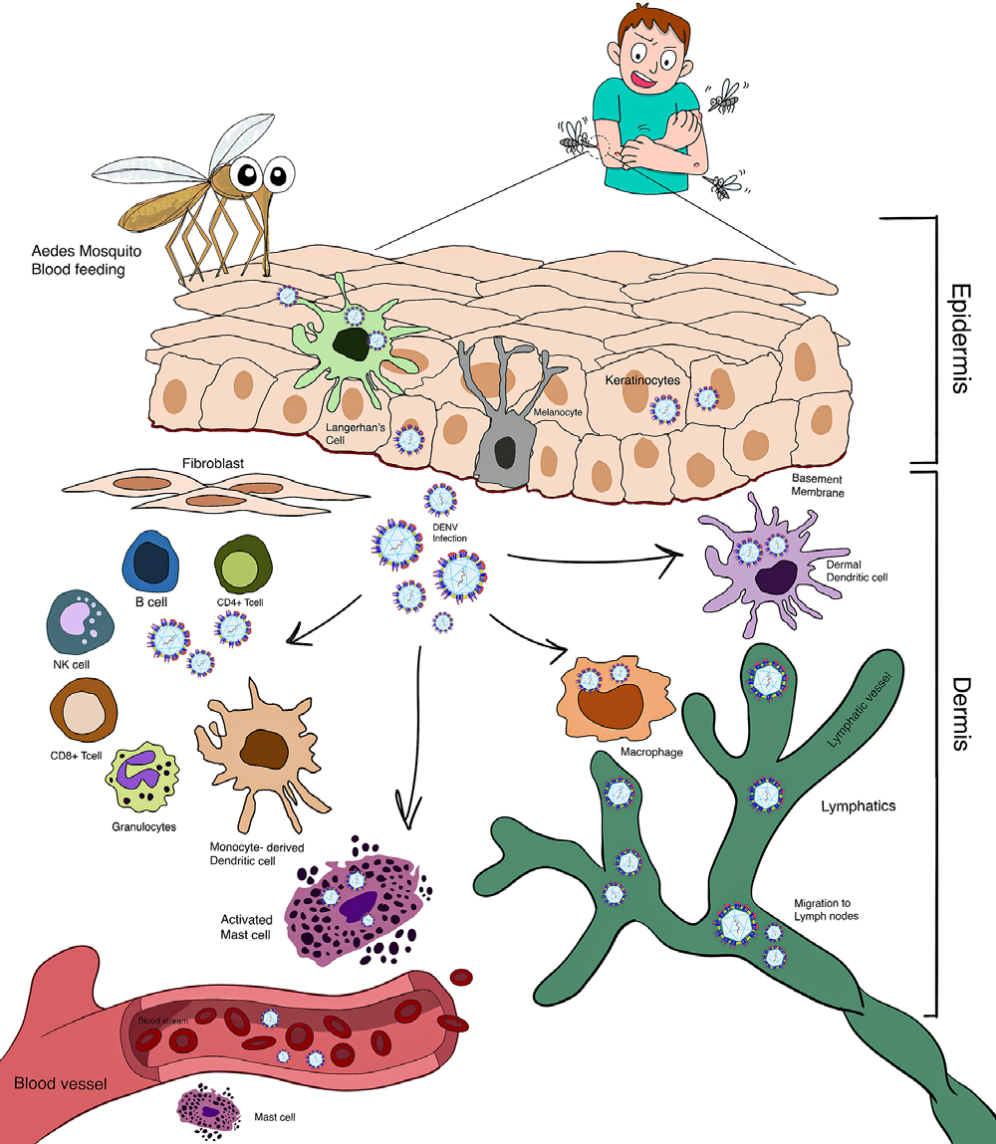
A pictorial representation of the immune response triggered in the skin during dengue infection. The diagram displays the network of immune cell types that encounter DENV in the skin during the early hours after infection. Few viruses are thought to be introduced into the epidermis through natural route infection; however, keratinocytes within the epidermis are one of the primary targets for viral dissemination. DCs, monocytes and macrophages are identified as the infection targets in the dermis. Although MCs are not substantially infected in the skin, they undergo activation by DENV and degranulate. This activation leads to the recruitment of NK cells to the infection site. Skin-homing T cells also migrate to the infected skin sites. Using lymphatics, infected DCs transport DENV to the draining lymph nodes.

The arbovirus transmission cycle is triggered when a vector ingests microbe-containing fluid during its blood feeding. These viral particles proliferate in the midgut and traverse to the salivary glands. Surprisingly, DENV induces multiple transcription factors that modulate viral replication in the salivary glands, which alter chemosensory attributes. This results in shaping bloodmeal selection by adjusting mosquito host-seeking or probing behaviour, which ultimately leads to increased viral transmission (Ref. 18). Female mosquitoes utilize a proboscis, which contains 1 × 10^4^–1 × 10^6^ plaque-forming units of the viral particle, that is, to be injected into the epidermis and dermis, thereby inducing an interaction between the pathogen and resident and migratory cells (Ref. 19). It is generally accepted that viruses are predominantly injected into the dermis; nevertheless, several investigations have indicated with respect to parasitic infection that pathogens are also found in the epidermis (Ref. 20), which is expected to also occur in arboviral infections owing to analogous saliva composition and probing behaviour (Ref. 19). DENV inoculum is rather minuscule; however, it is effective in efficiently invading the host (Ref. 19). The cells engaged during transmission have significant implications for the subsequent inflammatory cascade. A more thorough understanding of early responders, along with their ability to enhance replication and propagate, provides further insight into their pathophysiology (Figure 2).

## Keratinocytes

The skin, an intricate organ, executes diverse defensive roles against exogenous factors, and this protective role is accomplished by its structure, which comprises of three layers: the epidermis, the dermis and the hypodermis. These cells facilitate the pro-inflammatory and antiviral milieu created in the early stages of DENV infection. Consequently, the skin barrier harbours a diverse variety of indigenous and transient immunocompetent cells capable of guiding and triggering an effective immune response aimed at limiting pathogen propagation (Ref. 21). Keratinocytes contribute 90% to the integrity and infrastructure of the outer layer of the skin, suggesting the ability of viral particles to multiply in these resident cells constitutes a compelling prospect for host expansion. As previously reported, keratinocytes are more susceptible to DENV infection than dermal fibroblasts (Refs 21, 22). Pattern recognition receptors (PRRs) expressed in host cells, such as membrane-bound TLRs, nucleotide oligomerization domain (NOD)-like receptors (NLRs), protein kinase R (PKR), retinoic-acid-inducible gene I (RIG-I) and melanoma differentiation-associated gene 5 (MDA5), are the primary interactors in the immune recognition of DENV. Their activation results in eliciting an immune response (Refs 21, 22, 23, 24). These PRRs initiate signalling cascades by activating transcription factors such as nuclear factor kappa B (NF-κB), interferon (IFN) regulatory factor 3 (IRF3) and IRF7. Numerous IFN-stimulated genes (ISGs), proinflammatory cytokines and chemokines with varying degrees of antiviral activity are expressed as a consequence of activated IRF3 and IRF7 (Refs 23, 25).

**Figure 3.**
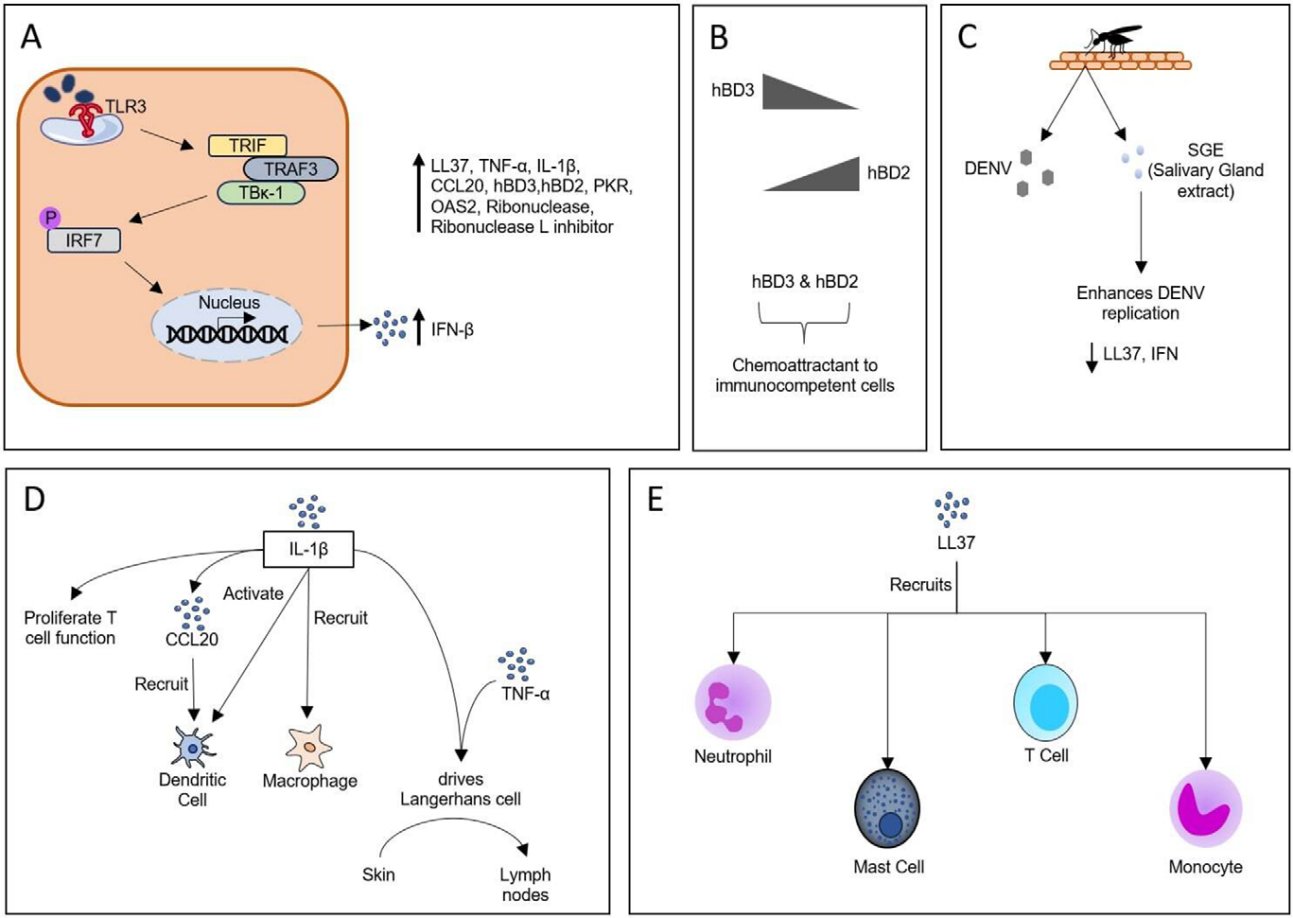
Immune response of keratinocytes. **A.** DENV is permissible to keratinocytes, which causes viral RNA to be sensed by intracellular TLR3, which then activates TRIF. Activated TRIF binds to TRAF3 and TBK-1, eventually leading to the activation of IRF7, which stimulates the synthesis of IFN-β. **B.** hBD3 expression heightens in the early stage of infection and gradually declines with an increase in the expression of hBD2. Both the antimicrobial peptides have chemoattractant properties. **C.** The pathogenic role of keratinocytes, SGE injected during blood feed, results in diminishing the expression of LL37 and IFN, thereby enhancing DENV proliferation. **D, E.** Schematic representation of the diversified role of IL-1β and LL37 in response to infection.

Keratinocytes function as an accessory cell in adaptive immune response and are one of the earliest targets, making up for 60% of all infected cells. As keratinocytes do not migrate, they are unable to aid in the dissemination of viral particles, but are equipped to secrete chemotactic factors that draw susceptible cells to the site of infection (Ref. 26). Surasombatpattan *et al.*(Ref 27) reported keratinocytes being permissive to DENV-2. The double-stranded viral RNA present intracellularly is sensed by TLR3, thereby exhibiting strong IFN beta (IFN-β) expression. Antimicrobial proteins, comprising human β defensin 2 (hBD2), hBD3, LL37 and RNase 7, known for triggering local innate response and attracting immunocompetent cells, become activated post-infection. Following dengue infection, hBD3 expression immediately spikes, but as this gradually declines, hBD2 expression peaks. Additionally, LL37 is a chemoattractant for mast cells (MCs), neutrophils, monocytes and T-cells, and shows enhanced expression following infection (Refs 27, 28, 29). IRF3 expression remained unaltered after DENV inoculation, whereas TLR3 and IRF7 expression increased. Other ISGs, including PKR, 2’-5’-oligoadenylate synthetase 2 (OAS2) and Ribonuclease L, are also activated, leading to heightened antiviral response. Another enhanced protein, Ribonuclease L inhibitors, counterbalance heightened immune response, thereby maintaining an equilibrium (Refs 27, 30). The pro-inflammatory factor interleukin-1beta (IL-1β) secreted by infected keratinocytes mediates the recruitment of macrophages, activates dendritic cells (DCs) and promotes T-cell function. Another function of IL-1β is that it induces C–C motif Chemokine ligand 20 (CCL20), another immunomodulatory factor, to recruit DC at the site of infection. Migration of Langerhans and dermal DCs from skin to lymph nodes requires IL-1β in conjunction with tumour necrosis factor alpha (TNF-α) for its dissemination (Refs 26, 28). As previously reported, salivary gland extract (SGE) is known to enhance DENV replication by suppressing LL37 and IFN titre, thereby resulting in viral evasion (Refs 29, 31). A study of heterologous infection highlighted that secondary dengue infection does not alter keratinocyte activation, indicating no probable role in the severity of infection (Ref. 32) (Figure 3).

## Mast cells

MCs, triggered by mosquito saliva, play an integral role in host defence because of their ability to regulate both immune responses. When infected, they secrete a variety of T_H_1-associated cytokines, along with histamine, leukotrienes, prostaglandins and chemokines, which are crucial for trafficking natural killer (NK) cells, monocytes and T cells (Refs 33, 34). Many of these compounds have been shown to activate endothelial cells, control the coagulation cascade and/or boost vascular permeability (Ref. 34). MCs have been reported to be degranulated in response to the viral components of DENV and are involved in DENV-induced vascular leakage. Post-degranulation, dengue viral components can be observed in the extracellular space or cytoplasmic granules. These granules have heparin and heparin-like glycosaminoglycans as their surface receptors, which also have a binding affinity for envelope protein. It implied that DENV can migrate in these extracellular granules from the initial site of infection to the lateral lymph node, another indispensable method for viral transmission (Ref. 35).

DENV-induced MC activation causes degranulation and increased secretion of inflammatory mediators such as IL-1β, IL-6, TNF-α, CCL3, CCL4, CCL5, chymase and histamine (Refs 34, 36, 37). Li *et al.* reported that salivary proteins, such as adenosine deaminase and al34K2, of *A. albopictus* alter the inflammatory response by interacting with MC-secreted proteases, known for inducing vascular leakage (Ref. 38). Similarly, chymase converts endothelin-1, a known factor involved in the pathogenesis of haemorrhagic shock, hypothesizing an associated response of MC and endothelial cells in dengue pathogenesis (Ref. 33). Multiple studies have documented chymase, a serine protease, to be significantly expressed in severe cases as compared to non-severe cases, suggesting its potential as a prognostic marker of severity (Refs 39, 40, 41, 42). A study conducted in Sri Lanka by Tissera *et al.* concluded chymase to be a more pronounced prognostic biomarker for children and adolescent patients as opposed to adult patients (Ref. 39). Additionally, they also reported that 96% of the severe cases were accurately predicted in the early stages of the infection (Ref. 39). Histamine, another protease found at high levels in dengue-infected patients, indicates its potential role in vascular leakage, which is known to contribute to severe outcomes (Refs 34, 36). The increased production of vascular endothelial growth factor (VEGF) following DENV infection aids in viral propagation by utilizing a lymphatic pathway to reach the draining lymph nodes. Furthermore, increased expression of VEGF and neuropilin inevitably leads to vascular leakage (Ref. 35). Localized expression is known to elevate cellular recruitment and vasodilation, which leads to DENV clearance, whereas systemic infection induces vascular leakage, culminating in DENV pathogenesis (Ref. 34).

**Figure 4.**
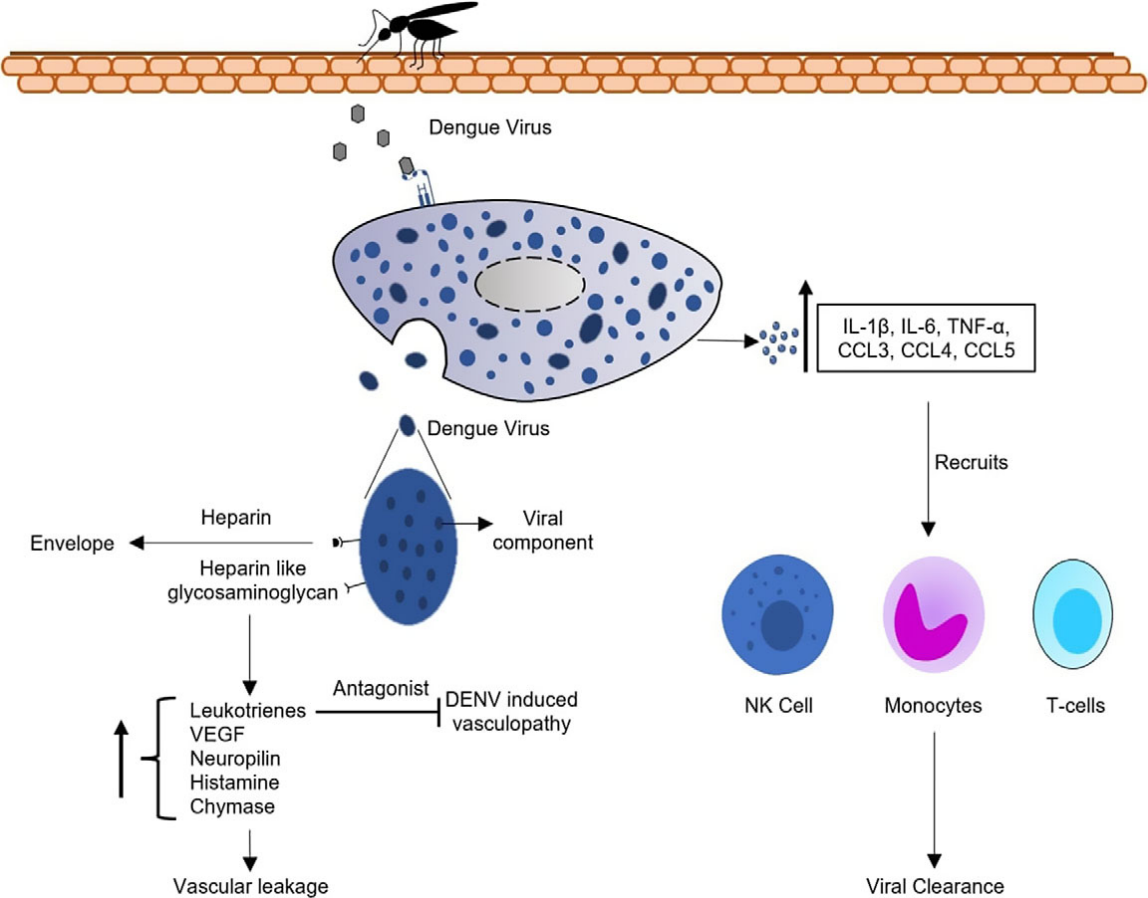
Protective or pathogenic role of mast cell in dengue infection. Viral interaction with receptors expressed on the surface of mast cells causes degranulation. DENV-induced degranulation secretes IL-1β, IL-6, TNF-α, CCL3, CCL4 and CCL5, which act as chemoattractants, recruiting monocytes, NK and T cells, essential for viral clearance. On the contrary, granules released during degranulation express heparin and heparin-like glycosaminoglycan on their surface, which has a binding affinity for envelope protein, resulting in viral dissemination. Other proteins secreted during degranulation, such as leukotrienes, VEGF, neuropilin, histamine and chymase, are responsible for altering endothelial permeability, resulting in vascular leakage.

In response to elevate the pathogenic role of MCs, Syenina *et al.* reported that MC-stabilizing medications could inhibit DENV-induced enhanced endothelial permeability *in vivo* and reverse uncontrolled pro-inflammatory expression (Ref. 33). Similarly, St John *et al.* hypothesized that utilizing a leukotriene receptor antagonist can be a therapeutic target for treating DENV-induced vasculopathy (Ref. 43). Thorough knowledge of MC-induced severe conditions might provide new perspectives on the development of therapeutics that target MCs for controlled degranulation (Figure 4).

## Dendritic cells

DENV infiltrates DCs via DC-specific intercellular adhesion molecule-3–grabbing nonintegrin (DC-SIGN) and Mannose Receptor (MR), which are the primary interacting receptors responsible for activation and for initiating the downstream signalling cascade by secreting TNF-α (Ref. 44). DCs are a type of specialized antigen-presenting cell comprising of three distinct subpopulations, which are known to play a crucial role in moulding the adaptive immune response. Immature myeloid DCs (mDCs) subsequently transform into an activated/mature state after being stung by a mosquito and secrete an array of cytokines and chemokines; additionally, they also migrate towards sites with a high concentration of T cells. Upon maturation, DCs exhibit diminished expression of DC-SIGN, highlighting their incompetence in getting infected by DENV. Upregulated expression of CD40, CD80, CD83 and CD86 was observed in infected and uninfected bystander DC cells. These upregulated CD markers facilitate interactions between infected and T cells (Refs 45, 46, 47). Activated DCs secrete enhanced titres of pro-inflammatory cytokines, which stimulate the expression of ISGs and apoptosis of epithelial cells (Ref. 47). IFN gamma (IFN-γ) circulating in the bloodstream could assist in promoting the maturation of immature DC by enhancing IL-12p70 expression, a known variable responsible for T_H_1 polarization (Ref. 45).

Glycosylated NS1 initiates the activation of TLR2/myeloid differentiation primary response gene 88 (MyD88) pathway, which subsequently promotes enhanced expression of inflammatory mediators. Moreover, during primary infection, DC maturation via TLR2/MyD88 activation results in T_H_2-based antibody response, whereas this shift in T-helper response leads to severe consequences during secondary dengue infection via ADE-mediated pathogenesis (Refs 48, 49, 50). TLR2 expression in DC and plasmacytoid DCs (pDCs) is more pronounced in severe cases as opposed to non-severe cases, hypothesizing their involvement in severe outcomes (Ref. 50). In contrast, recent studies have reported that TLR9, a receptor responsible for sensing DNA viruses, is activated during dengue infection. Upon dengue infection, the generation of reactive oxygen species (ROS) and inflammasome activation induced the release of mitochondrial DNA (mtDNA) in the cytosol triggering the activation of NF-κB and mitogen-activated protein kinase (MAPK) p38 signalling cascades, which are essential for eliciting antiviral responses. Additionally, TLR9^-^ exhibited a negative association with the innate immune response and viral load. A study conducted by Lai *et al.* reported that oxidized mtDNA was more pronounced in activating and upregulating TLR9 expression (Refs 50, 51). Similarly, Torres *et al.* reported that both pDCs and mDCs showed elevated expression of TLR9 in DF compared to dengue haemorrhagic fever (DHF), highlighting its role as an antiviral factor by triggering type I IFN response (Ref. 52).

A study by Sprokholt *et al.* concluded that post-24 h of inoculation, no host–virus interaction occurred, but viral RNA was necessary for activation. These activations were initiated by the cytoplasmic RIG-I and MDA5. DC-infected cell exhibited heightened expression of IL-1β, IL-6, TNF, IL-12p70, IL-8, C-X-C motif chemokine ligand 9 (CXCL-9), IFN-γ-inducible protein 10 (IP-10), CCL2, CCL3, CCL4 and CCL5. CCL2 and CCL4 aid in recruiting monocytes, while CCL3 potently attracts neutrophils to areas of inflammation. An auto feedback loop occurs in which DENV-infected DCs attract monocytes, upon their activation, results in recruiting additional DCs, thereby heightening the immune response (Refs 47, 53). Another research by Palmer *et al.* observed that DENV-infected DC had enhanced apoptotic gene expression, which also functioned as a stimulant for the bystander DC for maturation. Enhanced expression of IL-10 was additionally discovered, which might indicate the general rationale for suppression of immune response and viral clearance evasion (Ref. 54).

**Figure 5.**
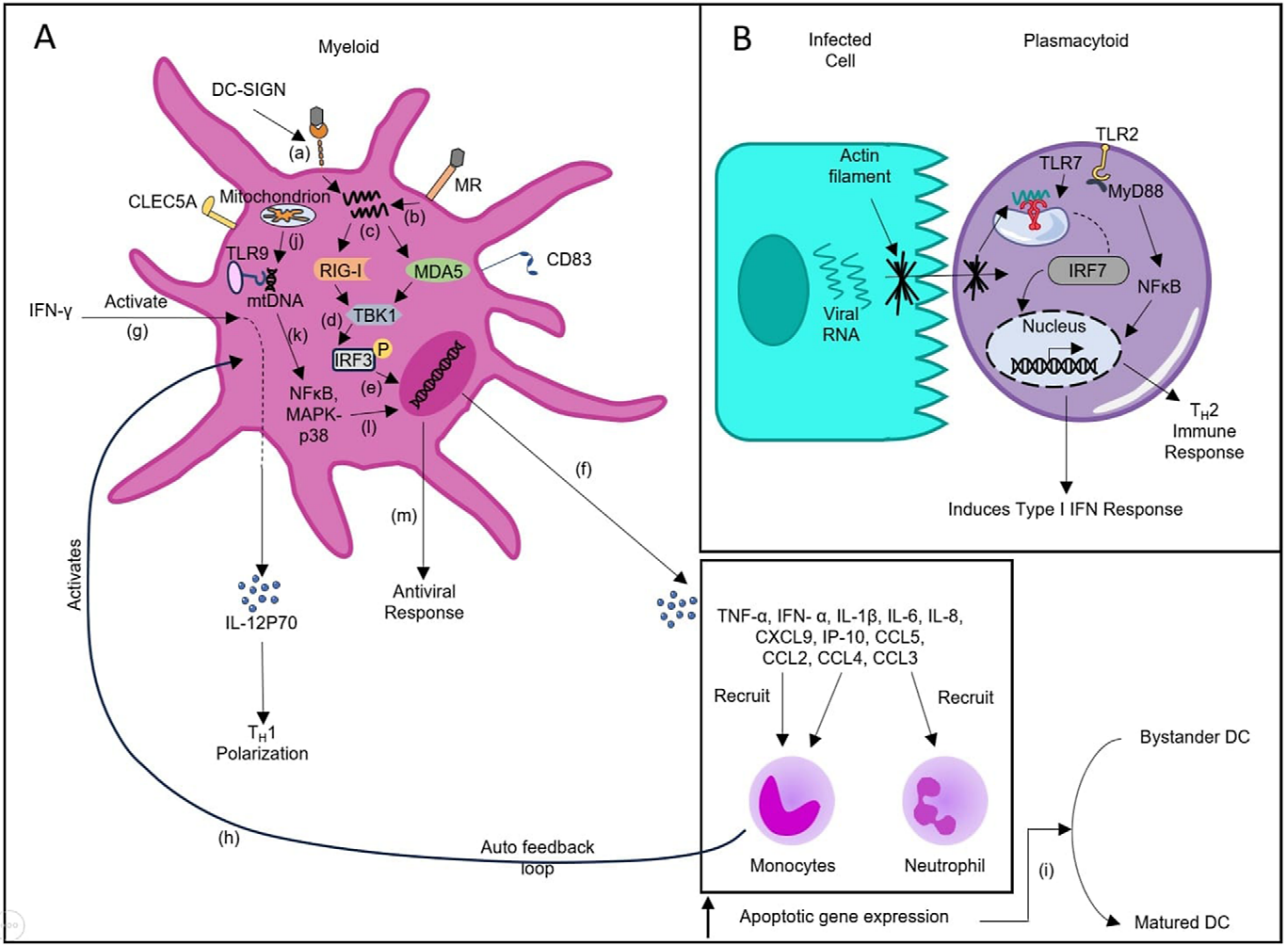
Dendritic cell in pathogenesis of dengue infection. **A. (a–f)** DENV is recognized by DC-SIGN and MR on the surface of myeloid dendritic cells, which is responsible for the internalization of the viral particle. This viral particle is sensed by RIG-1 and MDA5 in the cytoplasm, which induces type I IFN signalling cascade. Upon stimulation, they secrete TNF-α, IFN-α, IL-1β, IL-6, IL-8, CXCL9, IP-10, CCL5, CCL2, CCL4 and CCL3. CCL3 recruits neutrophils, and CCL2 and CCL4 recruit monocytes to the site of inflammation. **(h)** These monocytes subsequently, in an auto-feedback loop manner, activate dendritic cells, boosting the immune response. **(g)** IFN-γ in the circulation aids in inducing the expression of IL-12p70, which is essential for T_H_1 polarization. **(i)** As apoptotic gene expression increases, bystander dendritic cell gets activated, culminating in a heightened immune response. **(j–m)** Upon dengue infection, mtDNA released is sensed by the TLR9 receptor localized in the endosome, which initiates a cascade of events through the activation of NF-κB and MAPKp38 signalling, which is vital for eliciting an antiviral response. **B.** The mechanism by which plasmacytoid dendritic cells get activated is when a naïve cell interacts with a DENV-infected cell by forming a bridge-like structure composed of actin filaments. Viral particle traverses from the infected to the naïve cell via this framework. TLR7 on naïve cells interacts with viral RNA and induces type I IFN response. Additionally, another Toll-like receptor, TLR2/MyD88, present on the surface of dendritic cells, is crucial for triggering DC maturation and initiating a T_H_2-based immune response by activating the NF-κB signalling pathway.

pDC, a subpopulation of DC, is identified for secreting localized elevated levels of type I IFNs, recognize DENV in a distinctive mechanism by contact interaction. Following this interaction, viral RNA is sensed by TLR7 and ensures the activation of the signalling cascade, thereby suppressing the DENV infection (Ref. 55). A study by Gandhini *et al.* discovered that non-severe patients displayed a heightened frequency of activated circulating pDC indicated by enhanced expression of IFN alpha (IFN-α), and soluble TNF-related apoptosis-inducing ligand (sTRAIL) compared to severe patients, hypothesizing that activated pDC could potentially be utilized as a prognostic marker in the future (Ref. 56). Similarly, a study by Upasani *et al.* concluded two inferences: first, in severe patients, the frequency of circulating pDCs is reduced compared to DF patients; second, a link between IFN-α concentration and the ratio of circulating pDCs. This implies that this subset is solely responsible for type I IFN response and might probably impart protection from severe outcomes (Ref. 57). DENV has evolved multiple strategies to evade host response, one of which is inhibiting type I IFN response. Interestingly, this approach is ineffective for pDC, considering they autonomously activate the signalling cascade as no active viral replication occurs intracellularly (Ref. 58). A study conducted by Bittencourt *et al.* established that titres of pDCs and mDCs accumulated more in healthy individuals compared to non-severe adult cases, highlighting their modulation during infection (Ref. 59). Another research by Decembre *et al.* concluded that infected cells secreting immature viral particles enabled IFN-α response via pDCs more potently than cells efficiently producing completely mature virions, disclosing a functional role of immature viral particles in activating innate immunity (Ref. 60). High levels of DENV replication in mDCs have been reported, which coincides with DC-SIGN expression. On the contrary, diminished levels of DENV replication were noted in pDCs, but the inflammatory response in pDCs was more pronounced and rapid. The robust TNF-α and IFN-α response aids in regulating viral replication and, additionally, also interacts with other immune cell types, contributing to a pathogenic consequence (Ref. 61). Therefore, it can be concluded that both lineages of DCs serve their role in viral dissemination and inflammatory host response, thereby specifying their part in disease pathogenesis (Figure 5).

## Monocytes

Monocytes, one of the most prevalent blood mononuclear phagocytes, are the primary target cells of DENV. Post-interaction with the virus, they can differentiate into tissue macrophages and DCs (Ref. 62). They display an array of sensory receptors, such as TLRs, RIG-1 and MDA-5, which are known to be involved in pathogen recognition and stimulation of the inflammatory cascade (Ref. 63). Monocytes typically fall into three distinct subtypes depending on their phenotype and functionality. The aforementioned categories include the classical, intermediate and non-classical monocytes. The percentage of intermediate monocytes surges after infection during the initial phases and plasmablast levels at later stages, highlighting their contribution to the humoral response by stimulating resting B cells to mature forms (Ref. 62). Diminishing titres of monocytes is known to enhance systemic viremia. Additionally, they also act as the primary vessels of virus dissemination and inflammatory mediators, which disrupt the integrity of endothelial cells. However, they are equally effective in producing an antiviral response by secreting components that promote systemic viral surveillance. Emphasizing monocytes, like all other innate cells, have a bidirectional involvement with respect to pathogenesis and prophylaxis of the infection.

Wong *et al.* reported CD16^+^CD14^high^ cells were the primary secretors of inflammatory cytokines (IL-1β, TNF-α, IL-6, CCL2, CCL3 and CCL4) in response to DENV infection. Prolonged polarization or release of CD16+ monocytes into the circulation, in turn, culminates in severe consequences (Ref. 64). Furthermore, some of the cytokines secreted by monocytes following DENV infection, which include TNF-α, IL-1β and IL-8, have been reported to be an influencing factor for endothelial permeability (Ref. 65). Adikari *et al.* reported that NS1 has lipopolysaccharide (LPS) like activity. As previously established, LPS promotes IL-10 secretion via TLR4-mediated pathway. They proposed that secretion of IL-10, an anti-inflammatory cytokine from monocytes, might occur in a similar manner via the NS1-TLR4 pathway (Ref. 66).

Azeredo *et al.* reported a higher proportion of non-classical monocytes post DENV-2 infection, with a significant level of intercellular adhesion molecule (ICAM-1) -1 expression that serves as an inducement for propagation to the site of infection, along with TLR2 and TLR4 expression, which, on interaction with soluble NS1, as previously declared, activate NF-κB and IRF3 signalling cascade (Ref. 67). Aguilar-Briseño *et al.* disclosed similar findings, where enhanced levels of TLR2 associate with severe disease progression and decreased TLR2 levels correlate with symptoms of milder severity, thus tackling the TLR2 axis as a potential target that might mitigate the pathogenesis of severe disease by influencing vascular response (Ref. 68).

Arias *et al.* reported increased levels of TNF-α, IL-12, IL-17, soluble suppression of tumorigenicity (sST2), sTRAIL and IL-6 in dengue-infected individuals when compared to healthy controls; when analysed based on severity, IL-6 and sST2 exhibited a pathogenic role, and IL-12 and sTRAIL a protective role (Ref. 69). Marinho *et al.* pitched obstructing CR3 signalling inhibited 30% of the viral infection. In addition, they reported diminished expression of CR3, CR4 and CD59 with elevated expression of sC5b-9 in dengue-infected patients, inferring that this modulation is associated with severe consequences (Ref. 70). Moreover, Silva *et al.* reported that phospholipase A_2_ (PLA_2_) enzymes, prostaglandins and inflammatory mediators, which are released from activated monocytes, are pathogenic factors influencing vascular permeability (Ref. 71).

According to Corrêa *et al.* extracellular adenosine triphosphate (ATP) via the P2X7R pathway aids in diminishing NS1-infected cells, which ultimately influences the antiviral and inflammatory process. The newly discovered mechanism involving purinergic signalling in the backdrop of DF still requires investigation and could potentially be deployed as a prospective target for therapies (Ref. 72). Alhoot *et* *al.* further advanced the research about the activated surface receptor of monocytes in dengue infection by targeting CD14-associated molecules, known targets of DENV-2 entry. These effectively inhibited the DENV entry and subsequent virus multiplication, assisting as a novel target for inhibiting progression to severe outcomes as well as a promising therapeutic agent for the attenuation of dengue infection (Ref. 73).

**Figure 6.**
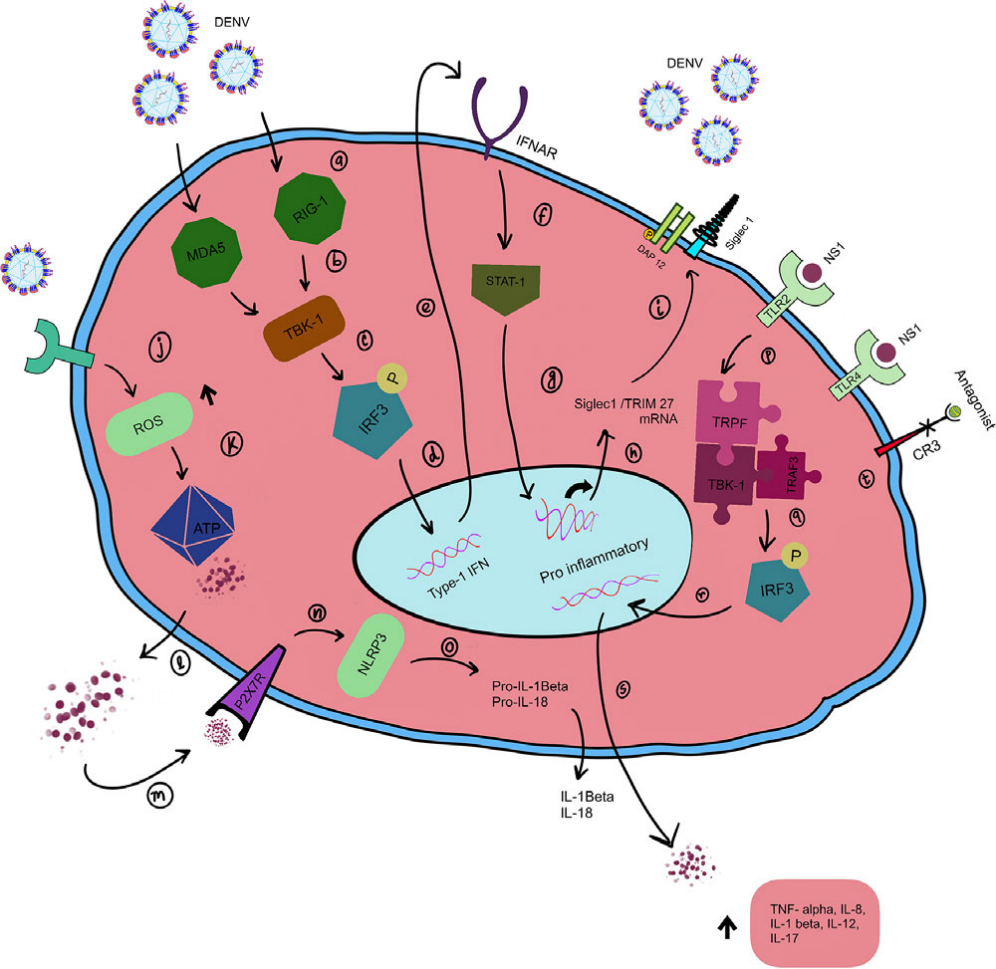
Role of monocytes in dengue infection. **(a–i)** DENV is sensed by intracellular RIG-1 and MDA5, which activate TBK-1. This interaction leads to the phosphorylation of IRF3. Activated IRF3 translocates to the nucleus and stimulates type I IFN synthesis. This response leads to the activation of IFNAR and subsequently STAT1, which induces the transcription of Siglec-1 and TRIM27 mRNA. Post-translational modification leads to siglec-1 being expressed on the surface of the cell and matured Trim27 degrades TBK-1, leading to impaired IRF3 signalling, hypothesizing a modulation of the immune response. **(j–o)** Another pathway by which monocytes aid in viral clearance is by activating the purinergic signalling. ROS generated in the cell induces ATP to be released from the cell; this extracellular ATP binds with P2X7R, which further activates NLRP3 inflammasome. This activation leads to the secretion of IL-1β and IL-18 into the circulation. **(p–s)** Soluble form of NS1 present in the circulation interacts with TLR2 and TLR4 and results in the activation of IRF3. Phosphorylated IRF3 translocates to the nucleus and triggers the synthesis of immune response genes such as TNF-α, IL-8, IL-1β, IL-12 and IL-17. **(t)** Blocking CR3 signalling reduced 30% of viral infection, a probable future therapeutic target.

Another adhesion molecule with dual roles is CD169, frequently referred to as sialic-acid immunoglobulin-like lectins (Siglec-1). In severe acute respiratory syndrome coronavirus 2 (SARS-CoV-2) infection, CD169 elicited a protective role (Ref. 74), whereas, upon trans viral attachment, it aided in the dissemination of the human deficiency virus (HIV) (Ref. 75). Fenutria *et al.*’s research revealed increased CD169 titres after dengue and ZIKA infections (Ref. 76). Similar to previously reported literature, high titres of sSiglec-1 were obtained in patients with DENV infection when compared to healthy controls (Ref. 77). Additional investigation into CD169 is essential and could potentially contribute to the development of a more effective prognostic or therapeutic target (Ref. 78) (Figure 6).

## Macrophages

One of the first target cells exposed to the DENV during blood feeding is the macrophage. Macrophages have been grouped into two fundamental classifications: pro-inflammatory M1 and anti-inflammatory M2. Macrophage polarization has been deemed to play a crucial role in illness manifestation and dissemination. Polarization into M1 phenotype is predicted to enhance inflammation and give rise to potent antiviral immune responses, whereas M2 macrophages are involved in tissue remodelling and immunosuppression (Ref. 79). Lee *et al.* discovered in their study that diminished M2 activation could expose individuals to bleeding risk and thrombocytopenia, potentially leading to severe dengue outcome, therefore hypothesizing M2 as a biomarker for distinguishing severe paediatric cases (Ref. 79). IFN-γ, secreted by lymphoid cells, is a principal mediator for the interaction between macrophages and lymphocytes. Granulocyte–macrophage colony-stimulating factor (GM-CSF) is one of the necessary factors for the alteration of dormant macrophages into M1 macrophages, which have an increased ability to present antigens and engulf pathogens and can recruit lymphocytes, NK cells and neutrophils at the site of infection (Refs 80–82).

The DENV NS1 in the bloodstream interacts with macrophages by engaging with TLR4, therefore inducing their stimulation and the release of mediators. Moreover, platelets that have been activated by NS1 via TLR4 form aggregates and are subjected to being engulfed by macrophages (Ref. 83). Furthermore, given the background of viral evasion, specific salivary components are involved in reducing the invasion of macrophages at the site of the bite. This reduction may be attributable to lower amounts of pro-IL-1β and CXCL2 at the bite site (Ref. 81). Similarly, Barros *et al.* reported that expression of SGE, a salivary component of *A. aegypti* minimized inducible nitric oxide synthase (iNOS) and proinflammatory expression, while enhancing IL-10 production. This interaction disrupted the polarization of M1 macrophages, without influencing the M2 polarization (Ref. 84).

Fink *et al.* described macrophages as one of the innate cells involved in limiting systemic viral transmission (Ref. 85). Previous studies by Züst *et al.* on a macrophage-type I IFN knockout model rendered mice susceptible to lethal DENV infection, highlighting their crucial role in triggering an efficient immune response (Ref. 86).

**Figure 7.**
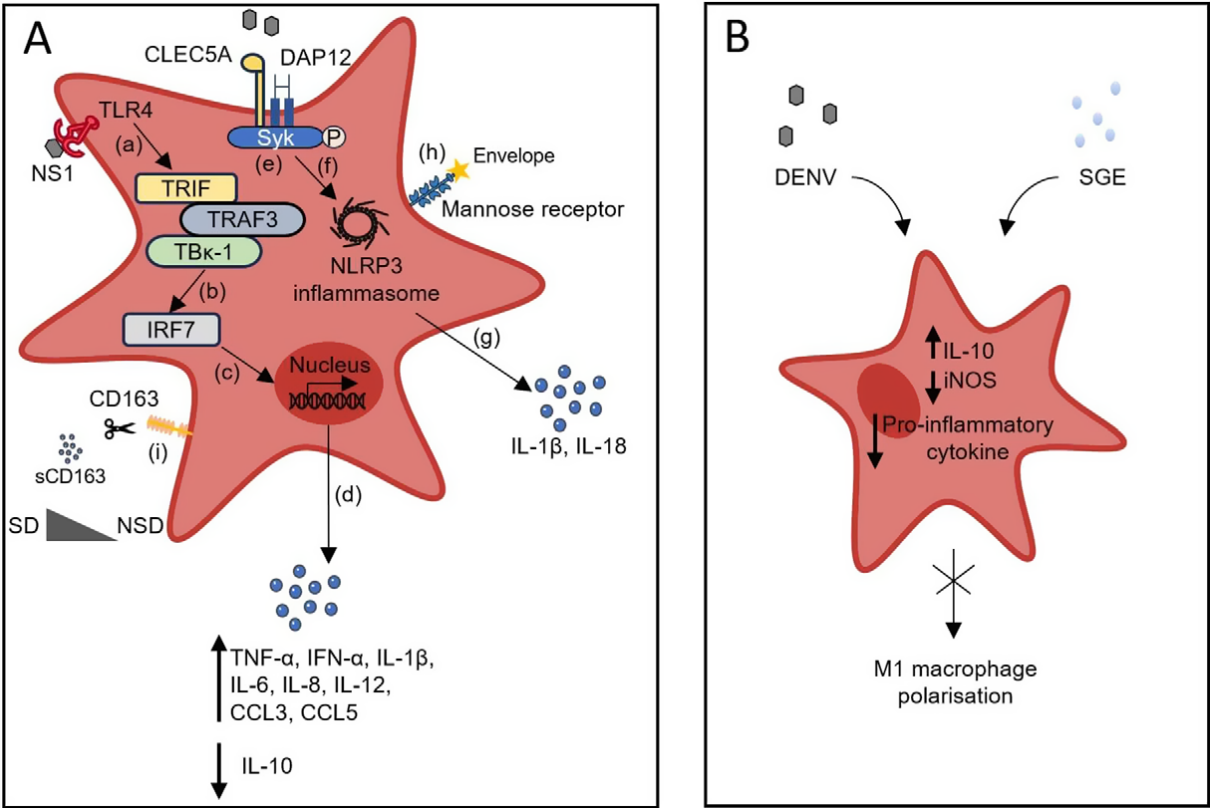
Macrophage role in dengue infection. **A.** **(a–d)** Circulating NS1 interacts with TLR4 expressed on the surface of macrophages, which in turn results in the activation of IRF7. Activated macrophage culminates in secreting TNF-α, IFN-α, IL-1β, IL-6, IL-8, IL-12, CCL3 and CCL5. **(e–g)** Another pathway by which macrophage gets stimulated is by interacting with CLEC5A with viral components. This interaction results in activation of the NLRP3 inflammasome, which causes IL-1β and IL-18 activation, which is essential for the induction of T_H_17 cells. **(h)** Mannose receptor interacts with envelope protein, aiding in viral internalization, which further modulates the immune response. **(i)** Upon infection, inflammatory cytokines trigger macrophages to express CD163, which, upon proteolytic cleavage, results in the formation of sCD163, a known disease severity marker. **B.** SGE secreted during blood feeding promotes downregulation of iNOS and pro-inflammatory cytokine expression and upregulation of IL-10; this signalling suppresses M1 macrophage polarization, which assists in the viral evasion mechanism.

Activated M1 macrophages secrete increased levels of pro-inflammatory mediators, such as TNF-α, IFN-α, IL-1β, IL-6, IL-8, IL-12, CCL3, CCL5 and reduced levels of IL-10 as a result, triggering a T_H_1 response (Refs 34, 80, 87). A separate investigation reported that M1 is more susceptible to DENV infection and yields an elevated level of IL-18 and IL-1β via C-type lectin domain family 5 member A (CLEC5A) stimulating the NLRP3 inflammasome pathway, thereby contributing to disease severity through heightened vascular permeability (Ref. 87). Independent of CLEC5A, another receptor crucial for viral uptake and attachment is MR on macrophages, which interacts with the envelope protein of DENV through its carbohydrate-recognition domain (Ref. 88). Studies done by Chen *et al.* on the dynamics of inflammatory mediators secreted by activated macrophages uncovered that following DENV infection, IL-8 production was most prominent, followed by that of CCL3 and CCL5. The levels of IFN-α, IL-12 and TNF-α were intermediary, whereas minimal levels of IL-1β were secreted (Ref. 89). In addition, upon dengue infection, proinflammatory cytokines, such as IL-6, TNF-α and IFN-γ, induce IL-10 secretion, which thereby results in triggering of macrophages expressing CD163 (Ref. 90). Moreover, CD163, a known marker of macrophage activation, is altered in multiple infectious diseases. A study conducted in the south-eastern part of India reported elevated but not statistically significant levels of soluble CD163 (sCD163) in severe dengue when compared to non-severe cases. Nonetheless, it showed a statistically significant difference when compared between primary and secondary dengue infection, with the latter being more pronounced, indicating its potential as a tool for monitoring disease progression (Ref. 91). In a similar context, another study conducted by Ab-Rahman *et al.* reported significantly elevated expression of sCD163 in severe cases when compared to non-severe cases (Ref. 90). This alteration might be due to the reduced no. of cases in the severe category. Similar to the previous report, Vuong *et al.*, in 2022, highlighted that sCD163 was elevated but not statistically significant in the severe leakage category when compared with the no and/or moderate leakage category (Ref. 92). More extensive studies with a larger sample size in the severe category are essential to denote sCD163 as a marker of disease prognosis (Figure 7).

## NK cells

NK cells are identified as innate, transient effectors having a requisite role in regulating the innate and adaptive immune responses following DENV infection. Both subgroups of NK cells, namely CD56^bright^ and CD56^dim^, exhibit distinctive roles in triggering an immune response, and the juxtaposition of different activation and inhibitory receptors possesses significance in NK-cell-mediated immune response. These immune cells secrete cytokines and cytotoxic proteins, including granzyme B and perforin. These secreted mediators then trigger an array of signalling events that aid in recruiting additional lymphocytes to combat against the infection (Ref. 93). DCs and macrophages are one of the primary targets of the DENV, which play an indispensable part in initiating the immune response. When these immune cells are infected with the DENV, they stimulate NK cells through two distinct mechanisms. The first mechanism involves the infected cells releasing immunomodulators, such as IL-12, IL-15, IL-18, TNF-α and type I IFNs. The second approach involves the interaction between the activatory or inhibitory receptors on NK cells and ligands present on the dengue-infected cells (Refs 94, 95).

Virus-infected cells commonly trigger or enhance the expression of ligands at their surface, allowing for the interaction between NK cell cytotoxic receptors, including NKp30, NKp44 and NKp46, and activatory or inhibitory receptors comprising activating killer cell immunoglobulin-like receptors (KIRs) and others, which interact with DENV peptides exhibited by human leukocyte antigen (HLA) molecules (Ref. 96). NK cells can discern cells infected with DENV by interacting with their activating receptors. However, the elevated levels of class I molecules of HLA may enable them to bypass the NK-cell-mediated immune response (Ref. 97). Previous studies highlight that KIR genes are in control of NK cell response; these findings indicate that the expression of certain KIRs on NK cells could systematically alter and influence the immune response (Ref. 96). Interaction between KIR3DL1 and HLA-B57, a conserved NS1 peptide, resulted in inhibition of NK cell activation; activated NK cells’ titre increases with reduction in viremia (Ref. 98). Similarly, interaction between HLA-C0102, a conserved NS3 peptide with KIR2DS2, and the envelope protein with NKp44 results in NK cell activation. On the contrary, KIR2DL1, another inhibitory receptor, interacts with DENV peptides presented by HLA-C molecules, and hinders NK cell activation (Ref. 96). Combining these research findings indicates that an intricate equilibrium is projected to exist between the activation and inhibition of NK cells, and they are likely to play a role in influencing NK cell responses, which culminates in having a functional status during the infection (Ref. 99). IL-18, often referred to as IFN-γ-inducing factor, is a cytokine capable of enhancing the production of IFN-γ by NK cells. Additionally, it leads to maturation and boosting the cytotoxic response. Occasionally, only the presence of IL-18 is adequate to induce IFN-γ production; however, in vivo conditions, IL-12 and IL-18 played a crucial role in ensuring the functionality of NK cells. It has been demonstrated that the IL-18 level spiked in cases of dengue infection, but revealed no association with NS1 or the infection type (Ref. 100).

**Figure 8.**
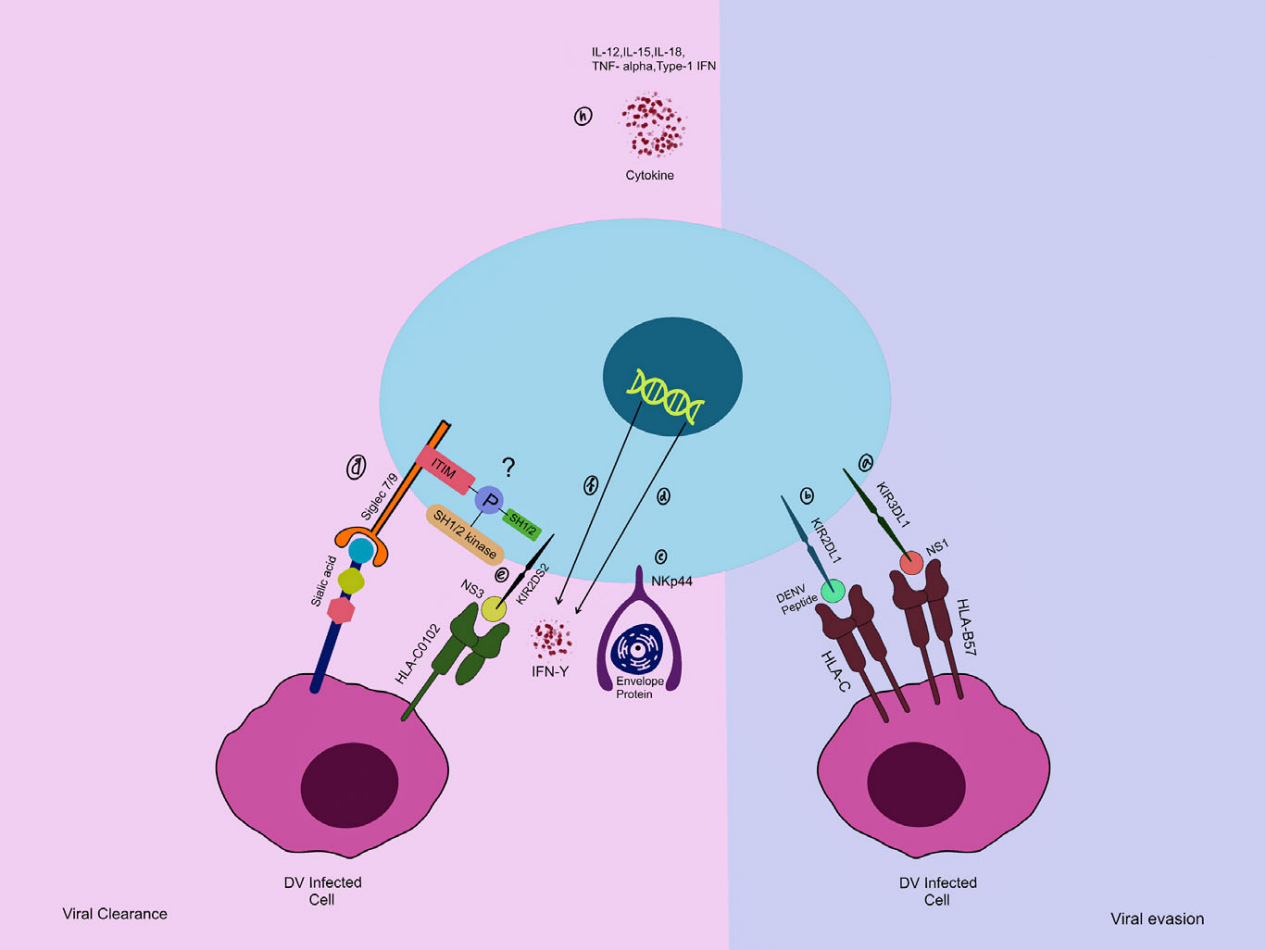
Interaction of dengue viral component with NK cell receptor and their role in pathophysiology. **(a, b)** Dengue NS1 presented by HLA-B57 suppresses the KIR3DL1 receptor on NK cell, an alternate receptor, KIR2DL1 on NK cells interacts with HLA-C, both the interaction further inhibits NK cell function, resulting in reduced cytotoxicity. **(c–f)** On the contrary, NKp44 interacts with the envelope protein, and KIR2DS2 binds with HLA-C0102, presenting NS3 leads to activation of NK cells, triggering enhanced IFN-γ expression, which leads to efficient viral clearance. **(g)** Siglec-7/9 interacts with sialic acid expressed on the target cell or pathogen itself; this interaction leads to phosphorylation of the ITIM region present at the cytosolic side. The phosphorylated ITIM recruits SHP-1 and SHP-2, which inhibit NK cell cytotoxicity. Its role in the modulation of dengue pathophysiology still remains to be explored. **(h)** An alternative pathway for NK cell activation is getting stimulation by IL-12, IL-15, IL-18, TNF-α and type I IFNs released by DENV-infected cells.

Siglec-7 (CD328) is an inhibitory receptor expressed on NK cells and is known for modulating cytotoxic signals. Diminished receptor expression and elevated circulating levels of siglec-7 in both HIV and Hepatitis C virus infections have been observed, highlighting it as an early indicator for NK dysfunctionality (Refs 101, 102). Siglec-9 (CD329), another inhibitory receptor on NK cells, is known to play a role in immunosurveillance. Enhanced levels of Siglec9+/NK+ cells were inversely related to viremia in HIV-infected individuals (Ref. 103). In another study, Zhao *et al.* reported that reduced siglec-9 titres were associated with enhanced HBV proliferation; moreover, inhibiting siglec-9 restored the NK cell functionality (Ref. 104). As the status of siglec-7 and -9 is being disclosed in other viral illnesses, it remains undetermined in one of the most widespread viral infections, that is, dengue. Combining current improvements on siglec-7/9 indicates their probable potential as future therapeutic targets in viral infections (Figure 8).

## Neutrophil

Neutrophils, a type of innate immune cell, are an essential component of the body’s defence mechanism. Their heterogeneity, prolonged response time and inadequacy in regulation contribute equally to beneficial and harmful effects. They have been extensively studied for their ability to undergo NETosis, a process by which they capture and render viruses inactive, hence restricting the viral dissemination. Additionally, they also have the ability to phagocytose viral components and secrete proteolytic enzymes, leading to enhanced inflammation, contributing to adverse consequences (Ref. 105). Multiple observations implied that neutrophils are not simply spectators during DENV infection; moreover, increased levels of pro-inflammatory mediators secreted by innate immune cells, such as IL-8 and TNF-α, could stimulate neutrophils. Additionally CCL3 is potent for chemoattracting neutrophils (Refs 53, 106, 107). In the study carried out by Rawat *et al.* they established that DENV-2 triggers CD16^bright^/CD62L^dim^ subtype of neutrophil, thereby unleashing mediators that serve a paradoxical role during dengue pathogenesis (Ref. 108). Similarly, proinflammatory mediators secreted, such as IL-8, IP-10 and CCL2, post-activation have been postulated to influence endothelial cell permeability (Ref. 109).

**Figure 9.**
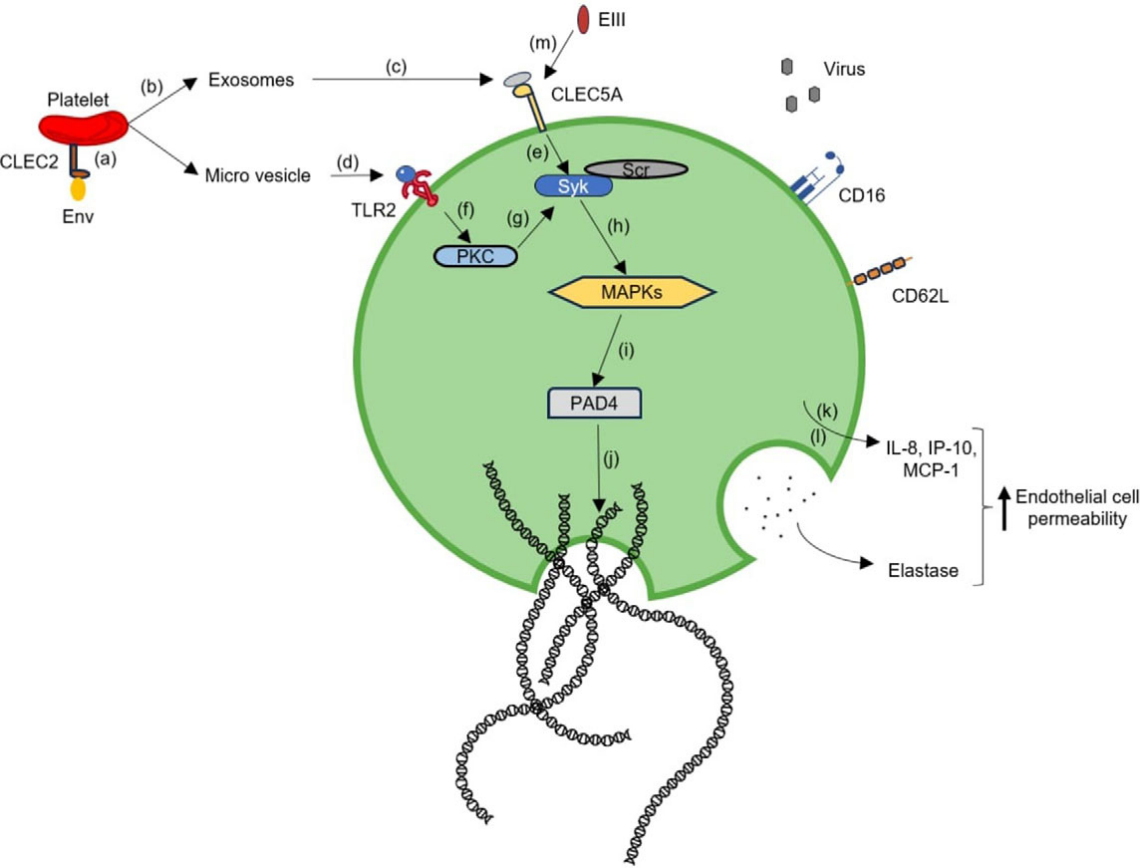
Overview of neutrophil in dengue pathogenesis. **(a–j)** DENV stimulates CLEC2 expressed on platelets to secrete exosomes and microvesicles. These platelet-derived microparticles, such as exosomes and microvesicles, interact with CLEC5A and TLR2 present on neutrophils, respectively. These receptors then induce MAPK signalling, which in turn activates PAD4. This culminates in, unfolding of chromatin and damaging the nuclear membrane, thereby enhancing DENV-induced NET formation. **(k-l)** Inflammatory mediators released by DENV-infected cells activate neutrophils, which results in the secretion of IL-8, IP-10 and MCP-1(CCL2). Upon activation, the neutrophil degranulates and elastase is released. Inflammatory mediators in conjunction with elastase enhance endothelial cell permeability. **(m)** Envelope protein domain III can also independently interact with CLEC5A to stimulate NET formation.

Research undertaken in Singapore, which enrolled 1,921 patients, concluded that only 11.8% of patients had neutropenia; a homogenous conclusion was reported by Agarwal *et al.* (Refs 110, 111). As previously observed, DENV infection facilitates degranulation and potential release of elastase, a well-researched variable accountable for endothelial barrier damage. They demonstrated that neutrophil elastase titres were higher in DHF patients when compared to DF, indicating their involvement in the inflammatory processes and their probable association with severity (Ref. 112). Opasawatchi *et* *al.* reported that priming by the pro-inflammatory milieu present during acute DENV infection activates neutrophils for ROS generation, which is an essential factor for ROS-dependent neutrophil extracellular trap (NET) formation. Additionally, they revealed that neutrophils, upon activation, are sensitive to delobulation, a preliminary factor of NET formation. They concluded that variables responsible for NET formation or disintegration play a crucial role in the outcomes of the infection (Ref. 106). Similarly, Moreno-Altamirano *et al.* revealed that DENV-2 suppressed phorbol 12-myristate 13-acetate-induced NET formation by 80% (Ref. 113). To broaden current understanding of NETs, Garishah *et al.* intended to identify the association between NETs and distinctive features of dengue, notably thrombocytopenia and endothelial dysfunction. Platelet-derived microparticles generated by platelets in response to DENV interaction stimulated the development of NETs via crosstalk with CLEC5A and TLR2 present on neutrophils. They uncovered that dengue infection correlates with NS1-dependent NOX-independent NET formation, which is associated with platelet activation, endothelial disruption and enhanced vascular permeability (Refs 114, 115). Similarly, Sung *et al.* reported that blocking CLEC5A or TLR2 reduced NET formation, thereby imparting protection against vascular leakage (Ref. 116). Lien *et al.* uncovered that envelop protein domain III unilaterally triggered neutrophil NETosis response *in vitro* and *in vivo*, and was additionally associated with cytokine storm, a known consequence of severe dengue (Ref. 117). Comprehending the precise mechanism responsible for NETosis and its protective or pathogenic impact on disease outcomes might provide perspective on feasible therapeutic approaches for DENV infections (Refs 106, 108) (Figure 9).

The interplay between the innate and adaptive immune response is one of the vital aspects of the immune response to pathogens. These innate lymphoid cells have numerous functions, including triggering adaptive immune response, which is essential for pathogen clearance, limiting severe consequences and developing memory cells, which might prove disadvantageous for subsequent infection.

During the initial stages of infection, the DENV infects innate cell, which, upon activation, secretes a distinct spectrum of inflammatory mediators for host defence. In addition, they amplify the infection by disseminating the infected cells to lymph nodes for further downstream immune cell activation. Moreover, infected cells, upon activation, also trigger the adaptive immune response (Ref. 45). A subset of DCs, that is, pDCs, secrete IL-6 and type I IFN, which promotes differentiation and activation of B cells (Ref. 118). Similarly, another subset of DCs, that is, mDCs, secrete IL-12 in the circulation, which are known stimulators of T-cell differentiation and cell-mediated immune response (Ref. 45). In conjunction with DCs, monocytes, NK cells, type I IFN and circulating cytokines are the main orchestrators of initiating the immune response, which aids in viral clearance.

The host response to invading pathogens is orchestrated by a complex network of innate cells and plasma proteins that are mainly represented by, but not limited to, the aforementioned axis.

Moreover, a concise summary of the differential expression of inflammatory mediators with respect to primary and secondary dengue infection, as well as the immune response to specific flavivirus infection, is delineated in the tables 1 and 2 listed below.

**Table 1.** Modification in plasma protein profile during primary and secondary dengue infection

Plasma protein	Primary dengue infection vs. secondary dengue infection
IL–1β	Higher expression level in secondary dengue as compared to primary dengue infection (Ref. 125)
TNF-α	Statistically non-significant difference (Ref. 119)
	Higher expression level in secondary dengue as compared to primary dengue infection (Ref. 125)
	Slight increase in expression level in secondary infection as opposed to primary infection (Ref. 126)
IL–6	Statistically non-significant difference (Refs 119, 126)
	Higher expression level in primary dengue as compared to secondary dengue infection (Ref. 121)
	Higher expression level in secondary dengue as compared to primary dengue infection (Ref. 123)
IL–12p70	Higher expression level in primary dengue as compared to secondary dengue infection (Refs 121, 126)
IFN-α	Higher expression level in primary dengue as compared to secondary dengue infection (Refs 119, 126)
IFN-γ	Higher expression level in secondary dengue as compared to primary dengue infection (Ref. 119)
	Statistically non-significant difference (Ref. 120)
	Higher expression level in primary dengue as compared to secondary dengue infection (Ref. 126)
CXCL9 (MIG)	Higher expression level in secondary dengue as compared to primary dengue infection (Ref. 120)
IP–10 (CXCL10)	Statistically non-significant difference (Ref. 120)
	Higher expression level in secondary dengue as compared to primary dengue infection (Refs 122, 124, 126)
IL–10	Statistically non-significant difference (Refs 119, 120)
	Higher expression level in secondary dengue as compared to primary dengue infection (Ref. 126)
IL–8 (CXCL8)	Statistically non-significant difference (Ref. 120)
IL–18	Statistically non-significant difference (Ref. 120)
IL–17A	Statistically non-significant difference (Ref. 119)
CCL2 (MCP–1)	Statistically non-significant difference (Refs 119, 120)
	Higher expression level in primary dengue as compared to secondary dengue infection (Ref. 126)
CCL3 (MIP–1α)	Statistically non-significant difference (Ref. 120)
CCL4 (MIP–1β)	Statistically non-significant difference (Ref. 120)
	Higher expression level in secondary dengue as compared to primary dengue infection (Ref. 121)
CCL5 (RANTES)	Statistically non-significant difference (Ref. 120)
	Higher expression level in secondary dengue as compared to primary dengue infection (Ref. 107)
CCL20	Statistically non-significant difference (Ref. 120)

**Table 2.** Comparative analysis of PRRs and plasma protein secretion to flavivirus infections

Virus	Receptors involved in activating downstream signalling	Plasma proteins	References
Dengue virus (DENV)	TLR2, 3, 4, 7 and 9 RIG–1 and MDA–5 CLEC5A DC-SIGN	IL–1β, TNF-α, IL–6, IL–12p70, IL–8, IFN-α, IL–10, IFN-γ, IL–17, IL–18, CXCL9, IP–10, CCL2, CCL3, CCL4, CCL5	(Refs 27, 44, 47, 50, 53, 54, 67, 69, 107)
ZIKA virus (ZIKV)	TLR2, 3, 7 and 8 RIG–1 and MDA–5 AXL and TIM–1 DC-SIGN	IL–1β, IL–2, IL–4, IL–5, IL–6 IL–7, IL–9, IL–1ra, IL–10, IL–13, IL–17, CCL3, IP–10, CCL5, IFN-γ	(Refs 127–130)
Yellow fever virus (YFV)	TLR3 RIG–1	IFN-β, IL–8, TNF-α, CCL5	(Refs 131–133)
West Nile virus (WNV)	TLR3 and 7 RIG–1 and MDA–5	IL–6, IL–12, CCL20, CXCL1, CXCL2, IL–8, IP–10, TNF-α, IL–1β, IL–10, IL–33, CCL5, CCL8, IL–1α, IL–17A, CCL4	(Refs 134–136)
Japanese encephalitis virus (JEV)	TLR3, 4, 7 RIG–1 and MDA–5	CCL2, CCL5, TNF-α, IL–6, IL–2, IL–4, IL–10, IFN-γ, IL–17, IL–8, CXCL9, IP–10, CXCL13	(Refs 137–142)

## Current status of vaccine and therapeutic research

Several studies have demonstrated that dengue severity is regulated by numerous host factors, of which uncontrolled stimulation of inflammatory mediators is the key element. While there are vaccine candidates, no approved vaccine or therapeutics are available for treatment to date. Currently, four dengue vaccine types are in their phase trials, including the inactivated vaccine, subunit vaccine and DNA vaccines, and the remaining live attenuated vaccine, CYD-TDV, commonly referred to as Dengvaxia®, the only licensed vaccine, and others such as TV003/TV005 and DENVax, are currently in phase III. They stimulate immunological responses by targeting the viral components (Refs 143-144). Dengvaxia® has two significant hurdles: it is restricted to being given to seropositive individuals over the age of 9 years, as it manifests increased likelihood of severe outcomes in seronegative individuals, and its weak efficacy towards the DENV-2 serotype (Refs 145–147). Since there are gaps in the understanding of immune response and pathophysiology of severe conditions, thereby impedes the development of DENV vaccines. Careful investigation of DENV-induced immune responses might be able to assist in the refinement of already developed dengue vaccines and propose alternative and/or heterologous strategies.

A tabular representation of different types of vaccines, their formulation and efficacy is mentioned in Table 3.

**Table 3.** Different types of DENV vaccine and their formulation, phase, dosage and efficacy

Vaccine type	Key candidates	Formulation	Clinical trial phase	Dosage time	Efficacy	References
Live-attenuated vaccines	CYD-TDV (Dengvaxia®)	Substituting prM and envelope from 4 dengue serotypes to the yellow fever vaccine strain	Phase III licensed	3 dosages with 6 months apart	Efficacy for DHF, severe dengue and hospitalized cases were 88.5, 80.8 and 67.2%, respectively.	Ref. (148)
					25-month efficacy–60.3% (age group 2–16 years)	Ref. (149)
					Efficacy against hospitalization for seropositive and seronegative individuals was 0.75%; 0.38% (2–16 years; 9–16 years) and 3.06%; 1.57% (2–16 years; 9–16 years) respectively.	Ref. (150)
	TV003/TV005	Three genetically attenuated virus+ chimeric virus (30 gene deletions in 3′ UTR) forming weakened strains of DENV1, 3 and 4	Phase III	Single dose	2-year efficacy– 79.6% (naïve individuals) 89.2% (individuals with a history of dengue infection)	Ref. (151)
					Antibody response 74% with TV003 and 90% with TV005	Ref. (152)
	TAK–003 (DENVax)	Blend of attenuated DENV 2 and chimeric DENV1, 3 and 4 (replaced premembrane and envelope protein)	Phase III	0 and 3 months	Overall efficacy–80.9% (safety population) 80.2% virologically confirmed dengue (VCD) 95.4% hospitalized VCD 74.9% seronegative	(Refs 148, 153)
					17 months efficacy– 73.3% 76.1% for seropositive 66.2% for seronegative 90.4% for hospitalized VCD 85.9% for DHF DENV1–69.8% DENV2–95.1% DENV3–48.9% DENV4–51%	Ref. (154)
					3rd year efficacy 44.7% for VCD 70.8% for hospitalized VCD	Ref. (155)
Inactivated virus vaccines	DPIV	Tetravalent purified inactivated virus AS01E/ASO3B (adjuvant)	Phase I	0, 28 days	Dengue naïve adults exhibited neutralizing antibodies against all serotypes (antibody titres reduced over time))	Ref. (152)
Subunit vaccines	V180	Truncated protein (80% of envelope protein from all 4 serotypes)) Adjuvant: ISOCOMATRIX or alhydrogel	Phase III	0, 1 and 2 months	79.6% effective in preventing dengue	(Refs 148, 151)
DNA vaccines	D1ME100	Recombinant plasmid vector VR1012 encoding prM and envelope protein of DENV1	Phase I	0, 1 and 5 months High dose–5mg Low dose–1mg	41.6% exhibited an antibody response in the high dose category. No neutralizing antibody in the low dose category. (flavivirus naïve subjects))	(Refs 148, 151, 156)
	TVDV	Recombinant plasmid encoding prM and envelope protein of DENV1/2/3/4+Vaxfectin (Adjuvant)	Phase I	0, 30, 90 days Group 1: TVDV (1 mg) Group 2: TVDV (1 mg) +Vaxfectin Group 3: TVDV (2 mg) +Vaxfectin	Group 1: No antibody response, 70% IFN-γ T-cell response Group 2: Minimal antibody response, 50% IFN-γ T-cell response Group 3: Minimal antibody response, 79% IFN-γ T-cell response	(Refs 151, 157)

Furthermore, researchers have dedicated decades to the development of antiviral agents for dengue therapy, but have achieved limited progress. Antiviral therapy aims to minimize the viral load, thereby lowering the disease burden (Ref. 158). Currently, no animal models are capable of mimicking human dengue symptoms or clinical manifestations (Ref. 158). Non-human primates are seldom utilized for evaluating the efficacy of antivirals because of their high manufacturing costs and breeding challenges. Murine models, such as AG129, BALB/c and C57BL/6 mice, are frequently used to assess *in vivo* therapeutic effectiveness. Antiviral drugs against DENV have been developed using methodologies that target host attachment factors, DENV viral components and post-infection stages (Ref. 158).

Tomatidine, an antiviral compound, does not have a direct virucidal effect but is reported to inhibit all four serotypes by hampering the viral production stage (Ref. 159). Some of the common therapeutics targeted by the above-mentioned methodologies are listed in Table 4

**Table 4.** List of compounds involved in the antiviral effect in dengue infection

Compounds	Model system	Serotype	Response	Ref
Bovine Lactoferrin	Vero cells	DENV–1, 2, 3, 4	Inhibit viral production by interacting with host receptors necessary for viral attachment.	(Ref. 160)
Carbohydrate-binding agents	Raji/DC-SIGN^+^	DENV–1, 2, 3, 4	Broad-spectrum antiviral activity (targets host–viral interaction)	(Ref. 161)
Doxycycline, Rolitetracycline	BHK–21	DENV–2	Inhibition of DENV propagation	(Ref. 162)
RK–0404678	Vero cells	DENV–2	Inhibit viral replication	(Ref. 163)
Quinine	HepG2	DENV–1, 2, 3, 4	Inhibited DENV production in all four serotypes by hampering the downstream signalling post viral attachment	(Ref. 164)
Baicalein	Vero cells	DENV–2	Inhibits infiltration and intracellular replication stages	(Ref. 165)
Ribavirin + CpdA	A549	DENV–2	Ribavirin – Reduced DENV production, IL–6, TNF-α, IP–10 and CCL5 CpdA – Reduced IL–6, TNF-α and CCL5 Combined treatment – Enhanced reduction in viral production	(Ref. 166)
SB203580 p38 MAPK inhibitor	Human PBMC, monocytic THP–1, KU812,	DENV–2	Reduced DENV-induced proinflammatory mediators (TNF-α, IL–8 and CCL5)	(Ref. 167)
	AG129 mice		Reduced risk of HCT and lymphopenia, better survival potential	
AZD6244 MEK/ERK inhibitor	AG129 mice	DENV–2, DENV–3	Inhibited from acquiring DHF Diminished viral load Increased IL–1β and TNF-α	(Ref. 168)
Cepharanthine	K562	DENV–1,2,3,4	Reduced Envelope protein (inhibiting host-viral interaction) and viral production	(Ref. 169)
	A549			
			Reduced IL–6 titres	
Polyphenols (Quercetin, Fisetin)	U937-DC-SIGN Macrophages	DENV–2, DENV–3	Alter viral replication cycle via interacting with E, NS1, NS3 and NS5 Quercetin – Block IL–6 and TNF-α expression; Alters IL–10 and IFN-γ levels Fisetin – Inhibit IL–6; Downregulates TNF-α, IL–10 and IFN-γ titres	(Ref. 170)
ST–148	AG129 mice	DENV–2	Reduces viremia via inhibiting the viral replicative stage Diminished titres of TNF-α, CCL2 and IL–6; with no difference in IFN-γ levels (statistically non-significant))	(Ref. 171)
1-stearoyl–2-arachidonoyl phosphatidylinositol	A549, BHK–21, HepG2, Huh–7	DENV–1, 2, 3, 4	Limiting cytopathic effect and replication without hampering viral entry (all serotypes) Diminished CCL5, CCL20, CXCL8, IL–6 and IFN-β titres (DENV–2)	(Ref. 172)
Uncaria Tomentosa	Human monocyte enriched PBMLs	DENV–2	Significantly reduced TNF-α and IFN-α	(Ref. 173)
Alpha-Mangostin	HepG2	DENV–2	Inhibits the replicative stage and suppresses NF-κB-mediated inflammatory mediator synthesis of CCL5, IP–10, TNF-α and IL–6	(Ref. 174)
	HepG2, Huh7	DENV–1, 2, 3, 4	Complete inhibition of DENV–1 and 3; reduction in DENV–2 and 4 Diminished cytokine-chemokine titres (IL–6, TNF-α, IP–10, CCL4 and CCL5)	(Ref. 175)
Pimecrolimus	Vero, BHK–21, B6(Cg)-Ifnar1tm1.2Ees/J mice	DENV–2	Inhibit DENV2 internalization without hampering the viral binding	(Ref. 176)
ethyl-p-methoxycinnamate	HepG2, A549	DENV–1, 2, 3, 4	Reduced viral replication for all serotypes Reduced titres of IL–6, TNF-α, CCL5 and IP–10 (DENV–2)	(Ref. 177)
Chloroquine	U937	DENV–2	Limits pro-inflammatory mediators’ synthesis (IFN-β, IFN-γ, TNF-α, IL–6 and IL–12)	(Ref. 178)
Baicalin	Vero	DENV–2	Hampers the viral replicative cycle and disrupts the attachment stage	(Ref. 179)

.

Despite the enormous number of molecules that show antiviral activity *in vitro*, only a handful have been subsequently researched and evaluated in clinical trials; nonetheless, none of them have sufficient efficacy to be utilized as antiviral medications (Ref. 158).

Chen *et al.* hypothesized that as CLEC5A is involved in DENV-induced inflammatory signalling cascade, blocking CLEC5A attenuated the inflammation and exhibited a potential to act as a therapeutic target by blocking severe outcomes (Ref. 180). C–C chemokine receptor 5 (CCR5), a chemoattractant involved in recruiting mononuclear leukocytes at the site of infection, is required for DENV-2 replication and progression. In respect to that, Marques *et al.* demonstrated blocking of CCR5 resulted in poor DENV-2 replication in macrophages, indicating an alternative approach to therapeutics by targeting chemokine receptors (Ref. 181). These findings emphasize the need for ongoing studies to uncover novel targets and compounds that may assist in avoiding or diminishing DENV infection.

## Conclusion and future perspective

The increasing prevalence of dengue cases due to urbanization and climate change poses a significant burden; despite that, there is no vaccine or antivirals. Having a comprehensive understanding of the host’s innate immune response to infection is crucial for identifying key therapeutic targets.

CLEC5A, TLRs, RIG-I, MDA5 and DC-SIGN play a crucial role in activating a wide range of innate cells and regulating the downstream signalling cascade. Moreover, uncontrolled activation leads to viral dissemination and proliferation, prompting a future target for therapeutics. Another distinct plausible target for therapeutics might be exploring the type I IFN axis, which, upon activation, induces a protective mechanism. A precise equilibrium between protective and pathogenic responses must be maintained to attain favourable outcomes. Various research on therapeutics targeting host-viral interacting molecules has been undertaken, but unfortunately, none of them have been established as DENV-specific antivirals. One of the possibilities would be that the host response is a complex interplay of multiple innate cells, so targeting a single aspect of the inflammatory response might not provide sufficient protection. Utilizing combination therapies might be a probable alternative for maximizing efficacy and minimizing adverse effects. Further research is necessary to elucidate the mechanism underlying the innate immune response in identifying novel targets and markers for disease severity and monitoring.

In a similar line of reasoning, Siglecs can be a novel and obvious target for theragnostic, as in prior investigations, Siglec-6-specific chimeric antigenic receptor-T cell conferred anti-leukemia reactivity (Ref. 182), Siglec-15 has been reported as a prognostic marker for giant cell tumour of bone. Additionally, anti-Siglec-15 was utilized as a prophylactic therapy in juvenile osteoporosis (Refs 183, 184). sSiglec-1 and sSiglec-5 are known type-I IFN signatures for systemic lupus erythematosus and prognostic markers for colorectal cancer, respectively (Refs 185, 186). In the recent threat of SARS-CoV-2, the need for an antiviral guided the development of a Siglec-9 agonist, which inhibited neutrophilic hyperinflammation (Ref. 187). As previously reported, targeting the IFN axis might be a promising approach. On the same notion, Faunza *et al.* hypothesized that targeting the viral component responsible for disrupting IFN response can be a potential target for suppressing infection (Ref. 188). Moreover, incorporation of recent findings into the existing understanding of dengue pathogenesis could be beneficial in researching for more advanced therapeutic targets and medications.

**Figure 10.**
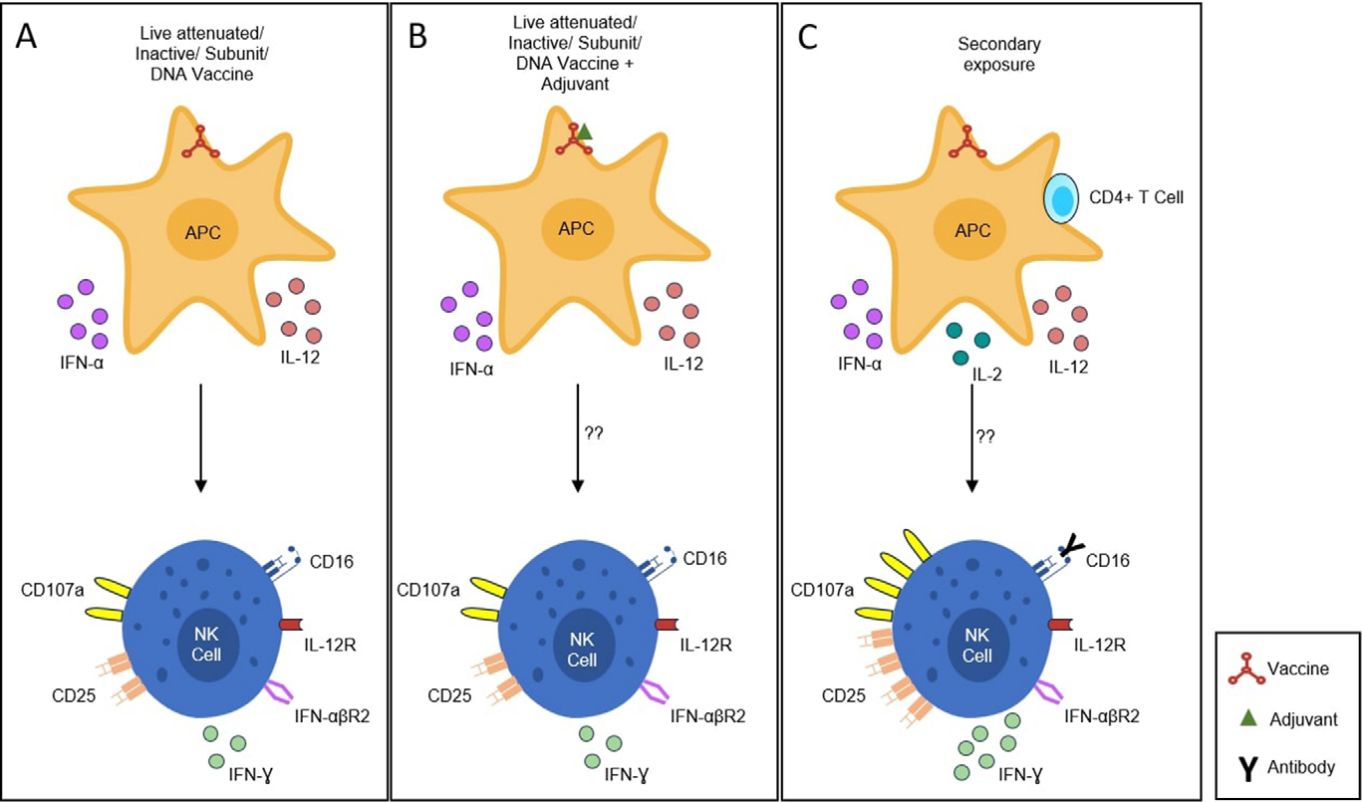
Hypothetical model of CIML NK cell in dengue vaccine research. **A.** Activation of APCs by vaccine could induce the release of cytokines which, in turn, might lead to the upregulation of CD107a and CD25, indicating increased NK cell cytotoxicity as well as differentiation into CIML-NK cell, respectively. **B.** Addition of adjuvants (AS03 / AS01?) in vaccine formulation might promote more production of CIML-NK cells, which in turn may stimulate more T and B cells along with IgG2a class switching. **C.** This formulation, on secondary exposure, might yield more enhanced protection and effectiveness.

Another aspect of advancing dengue vaccine studies is through stimulation of cytokine-induced memory-like (CIML) NK cells. CIML is a less differentiated subset of NK cells, which is known to have an enhanced ability to secrete IFN-γ. Wagstaffe *et al.* reported two significant points: one being the addition of adjuvants targeting the CIML population, manifested enhanced effectiveness, and the other being the difference in immunogenicity post vaccination might probably be due to the difference in CIML percentage, indicating CIML might potentially be a promising candidate for future translational dengue vaccine studies (Ref. 189). In-depth research is necessary for understanding the mechanism and potential benefits of CIML-NK cells in dengue vaccine research, and to determine whether incorporating CIML-NK cell-stimulating agents in dengue vaccine formulation brings advancement to existing vaccines (Figure 10).

## Abbreviations

ADE: antibody-dependent enhancement
ATP: adenosine triphosphate
CCL20: C–C motif chemokine ligand 20
CCL3: C–C motif chemokine ligand 3
CCL4: C–C motif chemokine ligand 4
CCL5: C–C motif chemokine ligand 5
CCR5: C-C chemokine receptor type 5
CLEC5A: C-type lectin domain family 5 member A
CXCL2: C-X-C motif chemokine ligand 2
CXCL9: C-X-C motif chemokine ligand 9
DC: dendritic cells
DC-SIGN: dendritic cell-specific intercellular adhesion molecule-3–grabbing nonintegrin
DENV: dengue virus
DF: dengue fever
DHF: dengue haemorrhagic fever
hBD2: human β defensin 2
hBD3: human β defensin 3
HBV: hepatitis B virus
HIV: human immunodeficiency virus
HLA: human leukocyte antigen
ICAM: intercellular adhesion molecule
IFN-α: interferonalpha
IFN-β: interferon beta
IFN-γ: interferon gamma
IL-10: interleukin-10
IL-12p70: interleukin-12 p70
IL-15: interleukin-15
IL-17: interleukin-17
IL-18: interleukin-18
IL-1β: interleukin-1 beta
IL-6: interleukin-6
IL-8: interleukin-8
iNOS: inducible nitric oxide synthase
IP-10: interferon-gamma-inducible protein 10
IRF-3: interferon regulatory factor 3
IRF-7: interferon regulatory factor 7
ISG: interferon-stimulated gene
KIRs: killer-cell immunoglobulin-like receptors
LPS: lipopolysaccharide
MAPK: mitogen-activated protein kinase
MC: mast cells
MDA-5: melanoma differentiation-associated gene 5
mDC: myeloid dendritic cell
MR: mannose receptor
mtDNA: mitochondrial DNA
MyD88: myeloid differentiation primary response gene 88
NET: neutrophil extracellular trap
NF-κB: nuclear factor kappa B
NK: natural killer
NKp30: natural cytotoxicity triggering receptor 3
NKp44: natural cytotoxicity triggering receptor 2
NKp46: natural cytotoxicity triggering receptor 1
NLR: nucleotide oligomerization domain(NOD)-like receptors
pDC: plasmacytoid DC
PKR: protein kinase R
PLA2: phospholipase A2
PRR: pattern recognition receptors
RIG-1: retinoicacid-inducible gene I
RNA: ribonucleic acid
ROS: reactive oxygen species
SC5b-9: soluble membrane attack complex
SGE: salivary gland extract
sST2: soluble suppression of tumorigenicity 2
sTRAIL: soluble TNF-related apoptosis-inducing ligand
TLR: Toll-like receptor
TLR2: Toll-like receptor 2
TNF-α: tumor necrosis factor-alpha
VEGF: vascular endothelial growth factor
WHO: World Health Organization
